# Latent profile analysis of health status and influencing factors among patients with coronary heart disease based on patient-reported outcomes

**DOI:** 10.3389/fcvm.2026.1739066

**Published:** 2026-01-23

**Authors:** Hong Jiang, Xiaochun He, Yuan Huang, Jingjing Tan, Xixi Li, Zhan Li

**Affiliations:** 1 Department of Cardiology, Mianyang Central Hospital, Mianyang, China; 2 Shenzhen Institution for Advanced Study, University of Electronic Science and Technology of China, Shenzhen, China

**Keywords:** chronic care management, coronary heart disease, health status classification, latent profile analysis, patient-reported outcomes

## Abstract

**Background:**

Coronary heart disease is a leading cause of mortality and disability worldwide, posing significant challenges to public health and necessitating effective strategies for improving patient outcomes and quality of life.This study aims to analyze the health status of Coronary heart disease patients using a patient-reported outcomes scale, exploring differences across five dimensions: physical health, mental health, social health, spiritual health, and specific symptoms. The goal is to provide a foundation for personalized medical interventions and health management.

**Methods:**

This is a Cross-sectional study, 240 patients were selected for latent profile analysis to categorize their health statuses. Key influencing factors were identified through univariate analysis and multivariate logistic regression analysis.

**Results:**

The health status of patients was categorized into three groups, stable and healthy model (*n* = 146), social psychological fluctuation model (*n* = 78), and symptom prominent instability model (*n* = 16). Significant differences were observed among these concerning glycated hemoglobin, high-density lipoprotein, low-density lipoprotein, age, monthly income, education level, and comorbid chronic obstructive pulmonary disease. The health status of social psychological fluctuation model and symptom prominent instability model was independently influenced by glycated hemoglobin, age, education level, and COPD (*P* < 0.05).

**Conclusion:**

The health status of CHD patients can be classified into distinct categories influenced by multiple factors and comorbidities. As a crucial assessment tool, PRO facilitates the categorization of patient health statuses and provides a reference for precision medicine and personalized interventions. Future efforts should focus on developing targeted interventions tailored to the specific characteristics.

## 1 Introduction

Coronary heart disease (CHD) remains a leading cause of death and disability worldwide. According to the Global Burden of Disease Study 2023, CHD accounted for approximately 8.91 million deaths globally, representing 14.8% of all deaths and ranking as the leading cause of mortality worldwide (1). CHD is characterized by a complex disease course, often accompanied by multiple chronic health conditions, which severely impact patients' physiological and psychological well-being. Recent studies indicate a continuous rise in the incidence of CHD among younger and middle-aged adults, reflecting a trend toward disease onset at earlier ages (2). This shift has been closely linked to metabolic abnormalities such as dyslipidemia, insulin resistance, and obesity, which are increasingly prevalent in this population. Beyond biological risk factors, social and behavioral determinants—including sedentary lifestyles, occupational stress, irregular work patterns, and chronic psychological distress—have been identified as critical contributors to CHD onset and progression (3, 4). In addition, socioeconomic disparities, particularly in education, income, and health literacy, significantly influence disease management, health behaviors, and access to high-quality care (5). These findings underscore the necessity of integrating epidemiological, psychosocial, and behavioral perspectives into CHD research to better understand patient heterogeneity and guide precision interventions.

Despite advances in medical technology that have improved survival rates, how to improving the quality of life and long-term health outcomes for these patients has become a focal point of clinical attention (6). Traditional clinical assessments have predominantly focused on biological indicators and objective outcomes, often neglecting patients’ subjective experiences and the complex relationships between their multidimensional health states. In recent years, increasing attention has been given to patient-centered care approaches, which recognize the value of incorporating patients’ perspectives into chronic disease management (7, 8). Patient-Reported Outcomes (PRO) as an assessment tool from a patient's perspective, by quantifying the patient's subjective feelings and assessing the impact of the disease on the patient's physical, psychological and social aspects, thereby complementing conventional measures and supporting individualized care (9, 10). The health status of patients with CHD has significant heterogeneity (11). However, most of the current studies on patients with CHD lack a comprehensive analysis of the multi-dimensional characteristics of health status, and rarely accurately stratify the patient population. Based on this, this study takes the PRO as the core, uses the LPA to identify potential health status profiles from multidimensional health data. Furthermore, the study explored the influencing factors associated with these profiles. The aim was to reveal the diversity of health status among CHD patients and its key driving factors, thereby providing theoretical support for precision management and individualized interventions, and offering a scientific basis for establishing a patient-centered management model for coronary heart disease.

## 2 Methods

### 2.1 Study design and participants

The primary aim of this study was to identify distinct health status profiles among patients with CHD based on patient-reported outcomes (PROs), and to explore the sociodemographic and clinical factors associated with these profiles.

This study employed a cross-sectional design and was conducted between November 2023 to June 2024 in three tertiary hospitals in Sichuan Province, China. Patients diagnosed with CHD were recruited through the cardiology inpatient departments. The study was approved by the hospital's ethics committee (Ethics Approval No: S202303120-01).

### 2.2 Research instruments

1. Patient Reported Outcome Instruments System for Chronic Disease-Coronary Heart Disease(PROISCD-CHD).This scale was developed by the team of Professor Zhang Chuanmeng. The Cronbach 's α coefficient of the scale was 0.89, including physical health (8 items). Mental health (8 items); social health (8 items); mental/belief health (6 items); there were 5 dimensions of specific symptoms and 45 items in the whole scale. Likertet grade scoring method was used to assign 1–5 points.
2. Patient basic information questionnaire: self-designed to collect the patient's age, gender, marital status, education level, occupation, family per capita monthly income and other basic information.
3. Clinical data record table of coronary heart disease: the patient's relevant clinical test indicators, drug types, and whether combined with other cardiovascular disease risk factors such as hypertension, diabetes, hyperlipidemia, etc.

### 2.3 Data collection procedures

Before distributing the questionnaires, researchers explained the purpose, procedures, and confidentiality principles of the study to ensure informed participation. Data collection took place during patients’ clinically stable period following hospital admission. Uniformly trained investigators conducted one-on-one questionnaire interviews in quiet inpatient wards to facilitate accurate and reliable responses.During the survey, investigators remained present to offer clarification and assistance as needed.

Participants did not receive any monetary compensation but were informed that participation could enhance their awareness of their own health status. Any missing or unclear responses were verified with the participant on-site to ensure data accuracy. Participants were reminded of the importance of providing honest and accurate responses to maintain the integrity and quality of the data.

### 2.4 Inclusion and exclusion criteria

Participants were eligible for inclusion if they met the following criteria: diagnosis of CHD confirmed by a cardiologist according to the Chinese Guidelines for the Diagnosis and Treatment of CHD, age ≥18 years; being conscious and able to communicate and comprehend basic information, with the ability to independently complete the questionnaire and voluntary participation with signed informed consent.

Exclusion criteria included severe cognitive impairment or communication barriers, acute myocardial infarction or other major cardiovascular events within the past three months, comorbid severe mental illness or malignant tumors or incomplete clinical or questionnaire data.

### 2.5 Statistical analysis

SPSS26.0 statistical software and R4.4.0 software were used for data analysis. LPA was performed using R software. LPA was conducted using the scores of the five dimensions of the PRO Scale as manifest variables. The model selection criteria included the following (12):

1. Akaik information criterion(AIC) and Bayesian information criterion(BIC) and sample-corrected Bayesian information criterion (aBIC), the smaller the value. It shows that the better the model fitting effect;
2. The classification index is the entropy value, and the value is 0∼1. The closer to 1, the higher the accuracy.
3. Likelihood ratio test indicators include the *P* value of LoMendell-Rubin likelihood ratio(LMR)and Bootstrap likelihood ratio test(BLRT). *P* < 0.05 indicates that the k-th category model is superior to the k-1-th category model.

For categorical variables, chi-square test was used for inter-group comparison; kmskal -Wallis H test was used for comparison of categorical data. For continuous variables, measurement data were expressed as $\left(\bar{x}\pm s\right)$ with normal distribution. One-way analysis of variance was used for comparison among multiple groups. If the data did not conform to the normal distribution, non-parametric test was used. After univariate analysis, variables with statistical significance (*P* < 0.05) were screened out, and then these variables were included in the multivariate model for multivariate analysis. Multivariate logistic regression analysis was used, and *P* < 0.05 was considered statistically significant.

## 3 Results

### 3.1 The total and each dimension score of PROISCD-CHD

A total of 240 questionnaires were distributed in this study, and 240 valid questionnaires were recovered, with an effective recovery rate of 100%. The score of 240 patients was (59.58 ± 0.81), and the scores of each dimension were physical health (64.14 ± 14.02), mental health (63.69 ± 14.59), social health (64.83 ± 14.27), mental belief health (45.80 ± 25.84) and specific symptoms (57.39 ± 19.18).

### 3.2 Latent profile analysis of the score of PROISCD-CHD

According to the scale scores of 240 patients with CHD, the LPA was carried out. The scores of five dimensions were used as explicit indicators. From the initial model, a total of five potential profile models were established, as shown in Table 1. According to the model screening criteria, although the fourth and fifth types of models AIC and BIC are lower, the entropy value is also lower. The third type of model not only has low model fitting AIC, BIC and aBIC values, but also has an entropy value of 0.98, showing high classification clarity, indicating that the classification of the model is more accurate. The significance test LMR and BLRT test *p* value is less than 0.05, indicating that it has a good fitting effect. Therefore, based on the above evaluation criteria, the 3 model performs better in fitting effect, classification clarity and statistical significance. it is more reasonable and more explanatory to choose model 3 as the optimal model.

**Table 1:** Fit statistics for the latent profile analysis.

Model	AIC	BIC	aBIC	entropy	LMR-p	BLRT-p	Class proportion
1	10,309.46	10,344.26	10,344.29	1	–	–	–
2	10,121.39	10,177.08	10,177.17	0.99	<0.001	0.01	0.93/0.07
**3**	**9,693**.**25**	**9,769**.**77**	**9,769**.**95**	**0**.**98**	**<0**.**001**	**0**.**01**	**0.64/0.29/0.07**
4	9,584.27	9,681.73	9,682.04	0.89	<0.001	0.01	0.30/0.36/0.28/0.07
5	9,470.68	9,589.02	9,589.50	0.92	<0.001	0.01	0.27/0.36/0.19/0.12/0.07

Boldface indicates the selected model.

**Figure 1:**
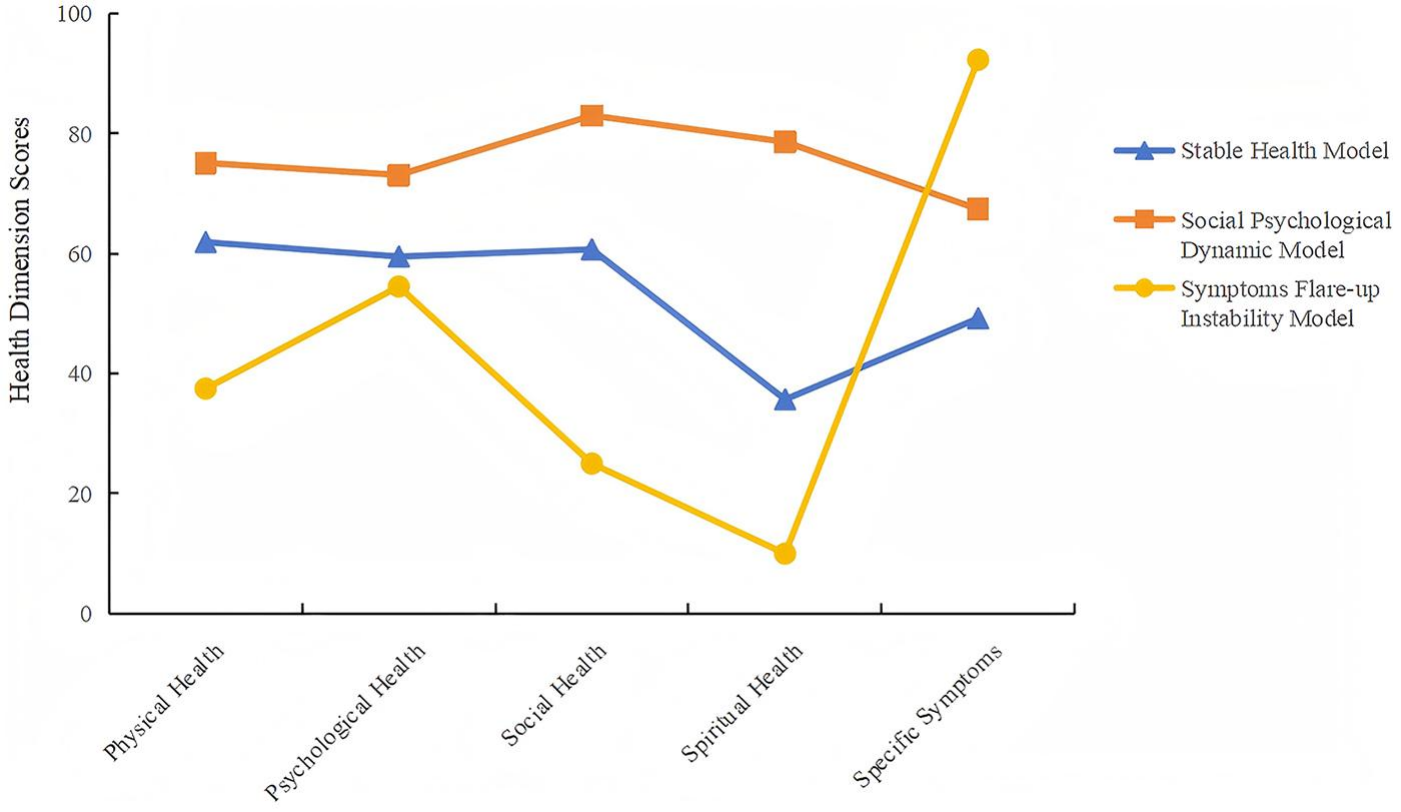
Latent profile indicators mean values for the three-profiles.

Each latent profile was named according to the scoring patterns in each dimension. Category 1 has less fluctuation in the five dimensions, so it is named Stable and Healthy Model (SHM). Category 2 has a higher score in the dimensions of mental health and social health, but there is a large fluctuation in the dimensions of mental belief health and specific symptoms, so it is named as Social Psychological fluctuation Model(SPFM). Category 3 has a very high score in the specific symptom dimension, while the scores in other dimensions are low and volatile, and the overall health status is unstable, so it is named as Symptom Prominent Instability model(SPIM). According to the scores of each dimension, a potential profile is made through the classification results, as shown in Figure 1.

### 3.3 Univariate analysis of latent classes

A total of 240 patients participated in the study. Among them, 146 individuals (60.83%) were classified into Latent Class 1, 78 individuals (32.50%) into Class 2, and 16 individuals (6.67%) into Class 3.A comparison of the three latent classes based on the PROISCD-CHD across various dimensions revealed statistically significant differences in glycated hemoglobin (HbA1c), high-density lipoprotein (HDL), low-density lipoprotein (LDL), age, monthly income, occupation, sex, type of medical insurance, number of medications, and the presence of comorbid chronic obstructive pulmonary disease (COPD) (*P* < 0.05). However, no statistically significant differences were observed for other variables (all *P* > 0.05), as detailed in Table 2.

**Table 2:** Latent Profile analysis of the score of PROISCD-CHD.

Variables	SHM (*n* = 146)	SPFM (*n* = 78)	SPIM (*n* = 16)	F/*χ*^2^	*P*
LVEF	58.97 ± 8.22	58.88 ± 8.43	57.00 ± 15.27	0.36	0.699^a^
TC	38.58 ± 414.86	4.15 ± 1.09	4.19 ± 1.66	0.32	0.725^a^
HbA1c	6.66 ± 1.41	7.04 ± 1.75	5.61 ± 1.98	5.75	0.004^a^
HDL	1.18 ± 0.59	1.18 ± 0.35	2.56 ± 5.54	6.45	0.002^a^
LDL	2.43 ± 1.18	2.40 ± 0.92	6.63 ± 17.15	6.71	0.001^a^
Cr	82.95 ± 36.76	80.52 ± 25.50	85.24 ± 28.67	0.21	0.814^a^
TnT	19.07 ± 45.16	35.83 ± 119.05	19.22 ± 21.15	1.25	0.288^a^
TG	3.09 ± 11.69	1.67 ± 0.94	8.45 ± 27.39	2.34	0.099^a^
ProBNP	191.53 ± 218.95	302.32 ± 511.39	183.27 ± 205.36	5.80	0.063^a^
Blood glucose	7.32 ± 2.52	8.35 ± 6.86	6.69 ± 2.07	1.75	0.176^a^
Age					
≤44	2 (1.37)	2 (2.56)	4 (25.00)	-	0.009^c^
45–59	54 (36.99)	22 (28.21)	5 (31.25)		
60–74	77 (52.74)	44 (56.41)	7 (43.75)		
≥75	13 (8.90)	10 (12.82)	0 (0.00)		
Gender				5.68	0.038^b^
Male	90 (64.38)	51 (65.38)	15 (93.75)		
Female	56 (35.62)	27 (34.62)	1 (6.25)		
Educational level				<0.001^c^	
Junior High School/lower	94 (64.38)	45 (57.69)	1 (6.25)		
High school or vocational	29 (19.86)	23 (29.49)	6 (37.50)		
College degree or above	23 (15.75)	10 (12.82)	9 (56.25)		
Marriage status					
Non-married	1 (0.68)	0 (0.00)	0 (0.00)		
Wedlock	133 (91.10)	73 (93.59)	15 (93.75)		
Divorced	2 (1.37)	1 (1.28)	0 (0.00)		
Widowed	10 (6.85)	4 (5.13)	1 (6.25)		
Occupation				5.26	0.044^b^
Manual workers	90 (60.96)	50 (58.97)	5 (31.25)		
Mental workers	56 (39.04)	28 (41.03)	11 (68.75)		
Monthly income(RMB)			14.13	0.007^b^	
≤3,000	37 (25.34)	18 (23.08)	1 (6.25)		
3,001–5,000	60 (41.10)	27 (34.62)	2 (12.50)		
>5,000	49 (33.56)	33 (42.31)	13 (81.25)		
Insurance			5.47	0.041^b^	
Resident insurance	76 (53.42)	52 (66.67)	12 (75.00)		
Employee insurance	70 (46.58)	26 (33.33)	4 (25.00)		
Physical Activity(min/day)			4.17	0.124^b^	
<30	80 (54.79)	53 (67.95)	8 (50.00)		
≥30	66 (45.21)	25 (32.05)	8 (50.00)		
Smoking status				1.01	0.603^b^
Smoker	71 (48.63)	40 (51.28)	6 (37.50)		
Non- Smoker	75 (51.37)	38 (48.72)	10 (62.50)		
Drinking status				1.02	0.601^b^
Drinker	83 (56.85)	44 (56.41)	7 (43.75)		
Non- Drinker	63 (43.15)	34 (43.59)	9 (56.25)		
Number of medication		-	0.0,481^c^		
≤5	48 (32.88)	33 (42.31)	4 (25.00)		
6	32 (21.92)	10 (12.82)	7 (43.75)		
7	28 (19.18)	17 (21.79)	0 (0.00)		
8	38 (26.03)	18 (23.08)	5 (31.25)		
Hypertension				3.35	0.187^b^
No	59 (40.41)	37 (47.44)	10 (62.50)		
Yes	87 (59.59)	41 (52.56)	6 (37.50)		
Diabetes				2.17	0.338^b^
No	86 (58.90)	43 (55.13)	12 (75.00)		
Yes	60 (41.10)	35 (44.87)	4 (25.00)		
COPD				12.66	0.002^b^
No	139 (95.21)	64 (82.05)	0 (0.00)		
Yes	7 (4.79)	14 (17.95)	16 (100.00)		
Number of diseased vessels				1.22	0.542^b^
≤1	72 (49.32)	42 (53.85)	10 (62.50)		
>1	74 (50.68)	36 (46.15)	6 (37.50)		

a Represents analysis of variance.

b Represents the chi-square test.

c Represents fisher exact test. LVEF, left ventricular ejection fraction; ProBNP, Pro-B-type natriuretic peptide; TNT, troponin T.

### 3.4 Logistic regression analysis of scores in CHD patients by category

An unordered multinomial logistic regression analysis was conducted using the categorical of the PROISCD-CHD scale as the dependent variable. Independent variables included those indicators that showed statistically significant differences in the univariate analysis, with the stable health type used as the reference group.Categorical variables were assigned values accordingly,as shown in Table 3.

**Table 3:** Coding of categorical variables.

Variable	Category	Code
Age (years)	≤44	1
	45–59	2
	60–74	3
	≥75	4
Education level	Junior high school or below	1
	High school or vocational school	2
	College degree or above	3
Gender	Male	1
	Female	2
Insurance type	Resident insurance	1
	Employee insurance	2
Occupation	Manual workers	1
	Mental workers	2
Monthly income (RMB)	≤3,000	1
	3,001–5,000	2
	≥5,000	3
Number of medications	≤5	1
	6	2
	7	3
	8	4

The results showed that HbA1c, age and combined COPD were independent risk factors affecting the health status of patients with social psychological fluctuation. Age and education level and not combined with COPD were independent influencing factors of Symptom Prominent Instability model (*P* < 0.05), as shown in Table 4.

**Table 4:** Multivariate logistic regression analysis of influencing factors of health status in patients with CHD.

independent variable	Social psychological fluctuation model				Symptom prominent instability model			
	β	*p*	OR	95% CI	β	*p*	OR	95% CI
HbA1c	0.228	0.024	1.256	1.03–1.532	−0.509	0.204	0.601	0.274–1.318
LDL	−0.065	0.675	0.937	0.691–1.270	0.047	0.806	1.049	0.718–1.531
HDL	0.171	0.628	1.187	0.594–2.369	−0.082	0.866	0.921	0.356–2.388
Age								
≤44	0.34	0.045	1.405	1.134–4.793	1.234	0.039	3.429	1.062–10.973
45–59	−0.265	0.645	0.767	0.249–2.368	1.425	0.516	4.159	3.547–5.862
60–74	−0.309	0.566	0.734	0.255–2.109	2.299	0.407	9.156	8.498–14.045
Gender								
Male	−0.048	0.889	0.953	0.486–1.869	1.605	0.202	4.980	0.422–5.798
Occupation								
Manual	0.148	0.665	1.159	0.593–2.265	−0.32	0.680	0.726	0.158–3.327
Educational level								
Junior High School/lower	0.607	0.251	1.834	0.651–5.165	−3.648	0.016	0.026	0.001–0.505
High school or vocational	0.63	0.245	1.877	0.649–5.429	−0.865	0.326	0.421	0.075–2.364
Monthly income(RMB)								
≤3,000	−0.436	0.387	0.647	0.241–1.737	0.58	0.688	1.786	0.105–30.288
3,001–5,000	−0.458	0.249	0.632	0.29–1.377	−0.264	0.791	0.768	0.108–5.436
Insurance								
Residents’ Health	0.61	0.121	1.841	0.851–3.985	0.003	0.998	1.003	0.136–7.406
Number of medication								
≤5	0.454	0.272	1.575	0.701–3.538	−1.056	0.253	0.348	0.057–2.128
6	−0.145	0.774	0.865	0.321–2.332	0.198	0.845	1.218	0.169–8.792
7	0.172	0.715	1.188	0.471–2.995	−10.015	0.854	4.471	1.557–1.284
COPD								
No	−1.731	0.001	0.177	0.062–0.504	−1.88	0.001	0.152	0.050–0.468

The reference category is: social stability. The age was ≥75 years old, the gender was female, the occupation was mental workers, the education level was college or above, the medical payment was based on the social security of the workers, the types of oral drugs were 8, and whether COPD was combined was used as a reference.

## 4 Discussions

### 4.1 Three types of potential profiles of health status in patients with CHD

Based on PRO, this study conducted a cross-sectional analysis of the health status of patients with CHD, and divided the health status into three categories (SHM. SPFM, SPIM).The proportion of SHM patients was the highest (60.83%). The scores of physiological, psychological, social and mental health dimensions were balanced, the overall fluctuation was small, the symptoms were mild, and the health status was stable, indicating that the health status of most patients was relatively stable, which was consistent with previous research results (13). These patients have higher scores in social health and mental health. Studies have shown that better mental health and social support networks, which can manage diseases effectively and maintain quality of life, reduce mortality and recurrence rates (14). Intervention strategies should focus on maintaining their health level, preventing degradation, and optimizing health behavior.

However, the health dimension scores of patients with SPFM, SPIM instability fluctuate greatly, suggesting that these patients may face more challenges in health management and need to develop more targeted intervention strategies. Patients with SPFM had higher scores in the dimensions of mental health and social health, but the dimensions of mental belief and specific symptoms fluctuated greatly. Despite having strong social support and psychological adaptability, the lack of spiritual health may limit their psychological resilience in coping with chronic illness. Studies have shown that the lack of spiritual belief health can easily lead to negative emotions such as anxiety and depression, and increase the burden of disease (15). Therefore, interventions for this profile should emphasize a combination of spiritual support and psychological counseling to enhance the patients’ disease adaptation abilities.

Patients with SPIM are the most serious in terms of specific symptoms, other health dimensions have lower scores, and the overall health status is poor. Such patients not only face significant physiological challenges, but also are accompanied by increased psychological burden and lack of social support. This dual physiological and psychological pressure may form a vicious circle, further exacerbating health instability. The multidimensional health indicators of these patients were significantly lower than those of other types, suggesting that religious beliefs or spiritual support may alleviate psychological stress and improve physiological symptoms, which is consistent with previous studies (16). Consequently, intervention should focus on symptom control, supplemented by multidisciplinary collaboration to improve mental health and social function.

### 4.2 Exploration of factors influencing health status in CHD patients

HbA1c level is an independent risk factor affecting the health status of patients with psychosocial fluctuation. The patients in this group had the highest level of HbA1c, suggesting that poor blood glucose control may increase the psychological burden and disease symptoms. Studies have shown that (17, 18) long-term poor glucose control can aggravate atherosclerosis and microvascular damage, further worsening specific symptoms and overall health levels. Significant differences were observed in HDL and LDL levels across the three profiles. The levels of HDL and LDL in patients with unstable symptoms were significantly increased, suggesting that lipid metabolism disorder is an important cause of unstable health. Although HDL is generally considered to be good cholesterol, high levels may reflect chronic inflammation and aggravate vascular damage (19); elevated LDL increases the risk of cardiovascular events by promoting atherosclerotic plaque formation and inflammatory response (20). Chinese blood lipid management guidelines (21) require that the primary goal should be to reduce LDL levels, combined with HDL functional assessment, to develop personalized interventions, including optimizing diet structure, increasing physical activity and alleviating psychological stress, in order to improve lipid metabolism disorders and improve patient health outcomes.

The influence of age and gender on health status is also significant, and the proportion of young and middle-aged patients (≤44 years old) in patients with prominent and unstable symptoms is higher. This may be related to greater occupational stress, unhealthy lifestyles, and underestimation of disease risk (22). However, due to the strong compensatory ability of young patients, the symptoms may be hidden but progress rapidly, which may lead to increased instability of health status. In addition, the proportion of males in this group is significantly higher, and studies have shown that male patients are more likely to show severe symptoms and unstable health status (23, 24).

The monthly income of patients with unstable symptoms was significantly higher than that of the other two groups, but high income did not directly translate into better health management. High-income people tend to be accompanied by greater professional responsibilities and stress, resulting in irregular work and rest and unhealthy eating habits, thereby offsetting the health benefits of income advantages (25, 26).At the same time, high-income groups may have higher expectations for physical health status. When health conditions are poor, they are prone to stronger psychological frustration and stress reactions, which affect disease management compliance (27). In terms of medical insurance payment methods, most patients rely on resident insurance, but the reimbursement ratio is low, which makes it difficult to meet the needs of high-quality medical services and affects the overall improvement of health status (28).

The data indicate that occupational type also shows significant differences in health status classification. Mental workers accounted for a significantly higher proportion of unstable symptoms. Studies have shown that (4), mental workers may lead to energy metabolism disorders and increased psychological stress due to long-term sedentary, high work stress and insufficient physical activity. These factors may lead to increased risk of coronary heart disease and decreased compliance with disease management. Moderate physical activity of manual workers may have a protective effect on cardiovascular health (29). For mental workers, intervention should be carried out by improving lifestyle, increasing physical activity and alleviating occupational stress; for manual workers, occupational health monitoring should be strengthened to avoid health risks caused by excessive physical activity.

The influence of education level on health status can not be ignored. Highly educated patients have a higher proportion of unstable symptoms. Studies have shown that (30), patients with high education have more cardiovascular risk factors, including overweight/obesity, diabetes, dyslipidemia, lack of exercise and unhealthy diet, which increase the risk of cardiovascular events. The age of first acute myocardial infarction is about 56.3 years old, about 10 years earlier than those with low education, which affects the stability of their health status. The type of medication is an important factor affecting the health status of patients.

The proportion of patients taking 6 or more drugs in unstable patients with prominent symptoms is higher. These patients may need to take more drugs because of the complexity of the disease and the severity of complications, which in turn may affect their treatment compliance and disease control. Studies have shown that (31), multi-drug therapy can effectively control cardiovascular risk through synergy, but too many types of drugs may increase the economic burden of patients, the complexity of medication, and the risk of drug interactions, which in turn affects the patient 's treatment compliance and disease control effect. Therefore, it is necessary to optimize the drug combination, streamline unnecessary multi-drug combination therapy, and strengthen medication education to help patients understand the importance of multi-drug treatment and improve medication compliance.

In addition, patients with COPD are more concentrated in the symptoms of prominent instability, suggesting that COPD has a significant negative impact on the health status of coronary heart disease. Dyspnea caused by COPD can increase the cardiopulmonary burden and form a vicious circle with coronary heart disease (32), resulting in a decline in the quality of life and increasing the complexity of health management. For patients with COPD, multidisciplinary collaborative management mode should be adopted to integrate cardiovascular and respiratory treatment, and mental health intervention and health education should be strengthened to improve their overall health status.

## 5 Study limitations

Although this study reveals the diversity of health status of patients with CHD from the perspective of patients, there are still some limitations. The sample is only from a single region and the sample size is relatively small and limits the generalizability of the findings. The representativeness of the sample is limited, and the external validity of the research results needs to be further verified. Future research can be carried out in a wider area to recruit more diverse sample groups.

## 6 Conclusion

This study divided the health status of patients with coronary heart disease into three model, highlights the multidimensional nature of health among patients with CHD, demonstrating that physical, psychological, and social dimensions are interrelated and collectively shape overall health status and its multi-dimensional influencing factors. By identifying latent health profiles based on PRO, our findings provide empirical support for an integrated conceptual model of health. Strengthening future interventions for patients with different health status need to develop personalized intervention strategies, especially for patients with prominent and unstable symptoms, and should focus on metabolic management, psychological support and social resource optimization.

## Data Availability

The raw data supporting the conclusions of this article will be made available by the authors, without undue reservation.
